# Location and functions of Inebriated in the *Drosophila* eye

**DOI:** 10.1242/bio.034926

**Published:** 2018-07-15

**Authors:** Janusz Borycz, Anna Ziegler, Jolanta A. Borycz, Guido Uhlenbrock, Daniel Tapken, Lucia Caceres, Michael Hollmann, Bernhard T. Hovemann, Ian A. Meinertzhagen

**Affiliations:** 1 Department of Psychology and Neuroscience, Dalhousie University, Halifax, NS B3H 4R2, Canada; 2 Department of Biology, Dalhousie University, Halifax, NS B3H 4R2, Canada; 3 Receptor Biochemistry, Faculty of Chemistry and Biochemistry, Ruhr University of Bochum, 44780 Bochum, Germany; 4 Molecular Cell Biochemistry, Ruhr University of Bochum, 44780 Bochum, Germany

**Keywords:** *Drosophila*, Histamine, Carcinine, Glycine, Transport, Electroretinogram, Phototaxis, Immunocytochemistry

## Abstract

Histamine (HA) is a neurotransmitter in arthropod photoreceptors. It is recycled via conjugation to β-alanine to form β-alanylhistamine (carcinine). Conjugation occurs in epithelial glia that surround photoreceptor terminals in the first optic neuropil, and carcinine (CA) is then transported back to photoreceptors and cleaved to liberate HA and β-alanine. The gene *Inebriated* (*Ine*) encodes an Na^+^/Cl^−^-dependent SLC6 family transporter translated as two protein isoforms, long (P1) and short (P2). Photoreceptors specifically express Ine-P2 whereas Ine-P1 is expressed in non-neuronal cells. Both *ine^1^* and *ine^3^* have significantly reduced head HA contents compared with wild type, and a smaller increase in head HA after drinking 1% CA. Similarly, uptake of 0.1% CA was reduced in *ine^1^* and *ine^3^* mutant synaptosomes, but increased by 90% and 84% respectively for fractions incubated in 0.05% β-Ala, compared with wild type. Screening potential substrates in Ine expressing *Xenopus* oocytes revealed very little response to carcinine and β-Ala but increased conductance with glycine. Both *ine^1^* and *ine^3^* mutant responses in light-dark phototaxis did not differ from wild-type. Collectively our results suggest that Inebriated functions in an adjunct role as a transporter to the previously reported carcinine transporter CarT.

## Abstract (precis)

**Summary:** *Inebriated* isoforms express in alternating glial layers of the *Drosophila* visual system, consistent with a role in histamine recycling in the eye, and its mutants exhibit blue light phototaxis defects.

## INTRODUCTION

Vision in *Drosophila* depends on recycling the neurotransmitter histamine (HA), which is released from the terminals of photoreceptor neurons (Hardie, 1987; Sarthy, 1989) in the first optic neuropile, or lamina, beneath the compound eye. For this, the enzyme Ebony, which is expressed in epithelial glia that surround the photoreceptor terminals (Richardt et al., 2002), conjugates the HA to β-alanine (β-Ala), to synthesize β-alanyl histamine (carcinine, CA). The enzyme Tan, expressed in photoreceptors (Aust et al., 2010), then hydrolyses CA to release its stored HA and β-Ala. In flies mutant for *tan*, the abnormal electroretinogram, lack of phototaxis and head HA content, which is reduced by 90% relative to wild type, all indicate that the fly's visual system depends strongly on the ability to retrieve HA from CA, through this recycling pathway (Hotta and Benzer, 1969; Borycz et al., 2002). In turn, the recycling pathway requires the action of transporters to shuttle histamine metabolites between photoreceptor and glial cells (Stuart et al., 2007). In addition, pigment cells in the overlying retina seem also to be involved in transporting some components (Borycz et al., 2012), indicating that the shuttle pathways are likely to be distributed and widespread.

We have recently reported that a novel CA transporter, CarT, is involved in CA reuptake into photoreceptors (Xu et al., 2015). Previously, another transporter – Inebriated (Ine) – had been proposed to act as a presynaptic transporter of CA (Gavin et al., 2007). While our results failed to support a function for Inebriated in the direct uptake of CA into the photoreceptor terminals in the lamina (Xu et al., 2015), they do not exclude a function for this transporter in carrying other elements of the HA recycling pathway or for the transport of CA between the layers of glia that enclose the lamina (Edwards and Meinertzhagen, 2010). This pathway may be essential to maintain a reserve of CA, which is stored in the layers of cells between the marginal glia that lie beneath the lamina and the fenestrated glia that overlie the lamina, and is situated beneath the basement membrane of the eye. This CA reserve could then be used to release its stored HA on demand. In vertebrates, multiple transporters expressed in glia and neurons commonly act to terminate signalling at synapses that use a range of neurotransmitters – except acetylcholine – and enable the recycling of fast neurotransmitters (Kinjo et al., 2013; Vandenberg and Ryan, 2013), raising the possibility that multiple CA transporters may likewise exist in the fly's eye. Our immunohistochemical findings for CA expression in the fly's eye (Borycz et al., 2012) support such a hypothesis, insofar as CA has been found in eye structures that do not themselves express CarT.

The *inebriated* (*ine*) mutation in *Drosophila* was first described by Stern and Ganetzky (1992), and a mutation named *rosA* involving the same gene was later reported by Burg et al. (1996). The gene encodes a Na^+^/Cl^−^-dependent neurotransmitter/osmolyte SLC6 family transporter and generates two protein isoforms, one 943 amino acids long (Ine-P1) and a shorter one (Ine-P2), with approximately 300 fewer amino acids (Huang et al., 2002). Earlier studies failed to confirm that Inebriated transports any substrate, however, and observed only its effect on the movement of Na^+^, K^+^ and Cl^−^ ions (Chiu et al., 2000; Huang et al., 2002; Huang and Stern, 2002). Our current studies using antibodies against the long and the short isoforms of Inebriated now reveal a highly organized non-overlapping distribution of each isoform within the eye. We also observed a reduction in the head content of histamine in *ine^1^* and *ine^3^* mutants, suggesting an action of the gene on some aspect of histamine transport. The present study was therefore undertaken in order to characterize and locate the action of Ine in *Drosophila*, in an effort to identify its actions in histamine recycling, and its outcome on vision.

## RESULTS

### Immunohistochemistry

**Fig. 1.**
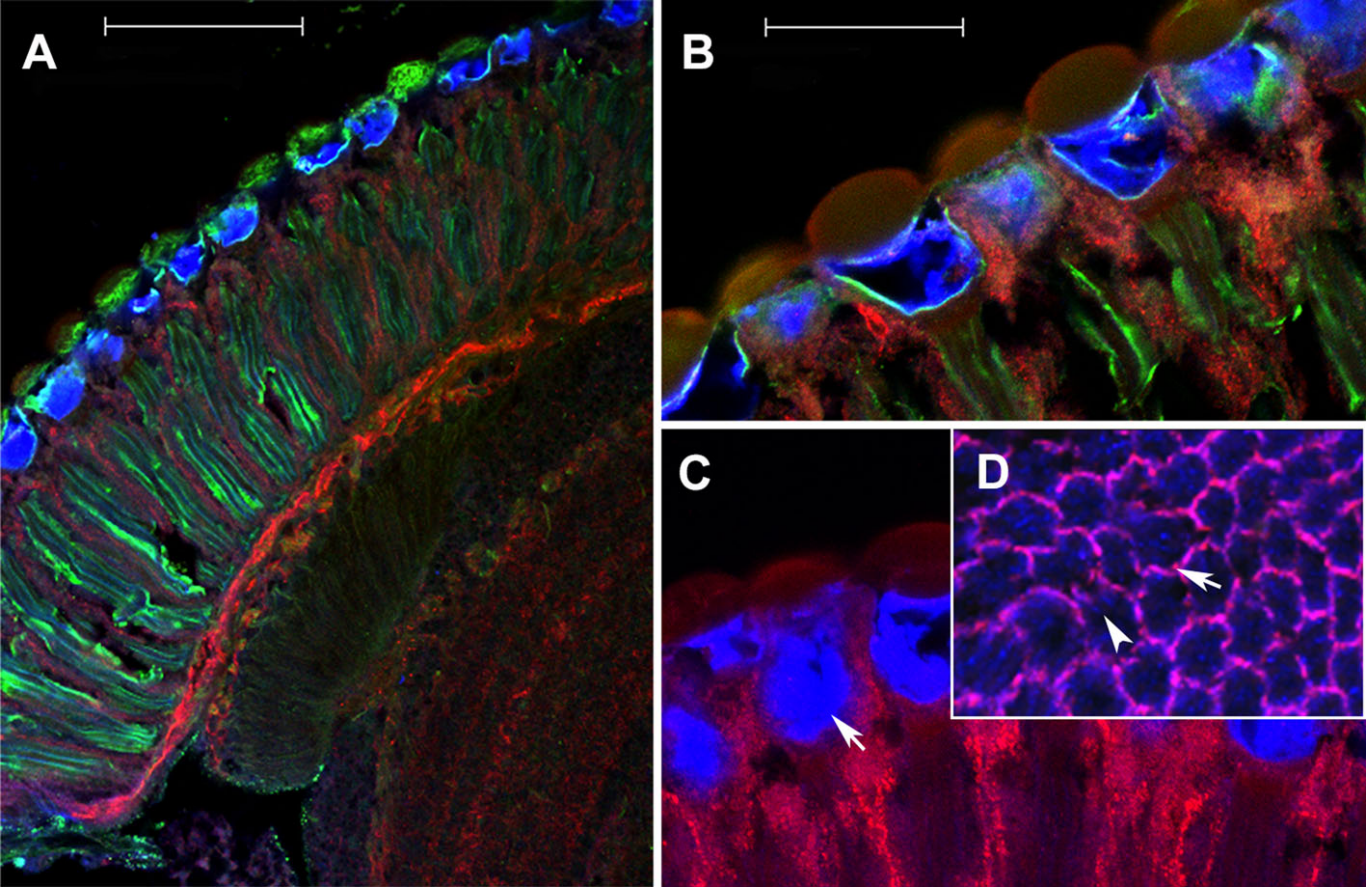
**Expression of P1 and P2 isoforms in the compound** **eye.** Oregon R wild-type flies labelled with antibodies against carcinine (green channel), and the P1 (red channel) and P2 (blue channel) protein isoforms of Inebriated. (A) In the compound eye P1 and P2 signals mostly fail to overlap. (B) Enlarged view of distal retina. (C) In the retina P2 (blue) strongly labels pseudocone cavities. (D) Inset: transversely sectioned ommatidia. P1 labels secondary and tertiary pigment cells (red, arrow) whereas P2 labels mostly photoreceptors (blue, arrowhead). Scale bars: 50 µm (A); 20 µm (B).

**Fig. 2.**
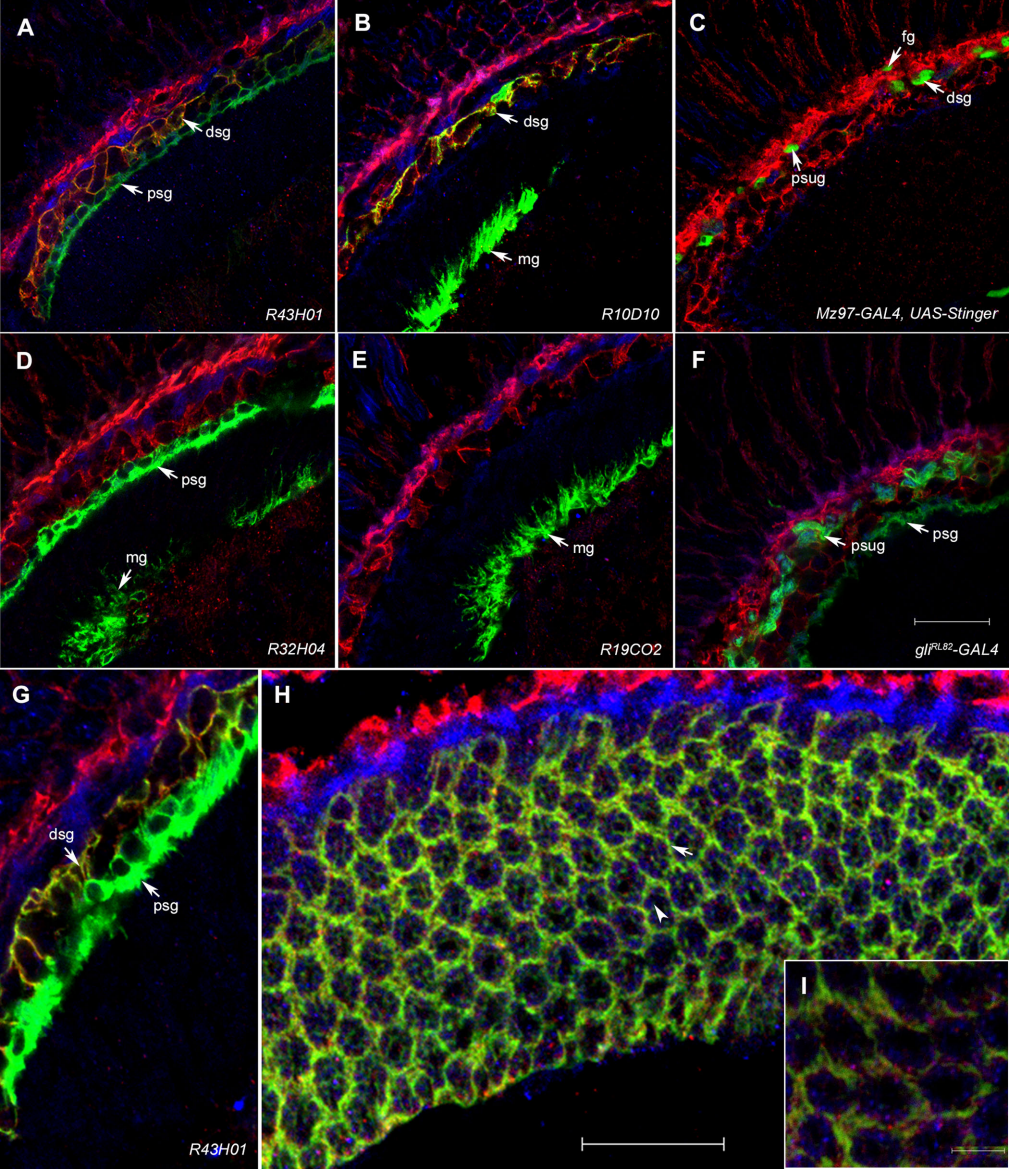
**Expression of P1 and P2 isoforms in the optic lobe.** Ine-P1 (A,B,C; magenta) and Ine-P2 (D,E,F; blue) co-expression with Gal4 driven GFP expression (green), reveals specific types of optic lobe glia (Edwards et al., 2012). (A) Ine-P1 co-expresses with GFP in the distal satellite glia (dsg) but not in the proximal satellite glia (psg). (B) Ine-P1 co-expresses with GFP in the distal satellite glia (dsg) but not in the marginal glia (mg). (C) Ine-P1 co-expresses with GFP in the pseudocartridge glia (psug). (D) Ine-P2 co-expresses with GFP in proximal satellite (psg) and marginal (mg) glia. (E) Ine-P2 co-expresses with GFP in marginal glia (mg). (F) Ine-P2 co-expresses with GFP in pseudocartridge (psug) and proximal satellite (psg) glia. (G) Ine-P1 co-expression with GFP in the distal satellite glia (dsg), enlarged from A. (H) Transverse section of lamina cartridges. Ine-P1 co-expresses with epithelial glia marker (arrow) and Ine-P2 expresses in the photoreceptors (arrowhead). (I) Enlarged view of cartridge array. Glia are labelled with the following abbreviations (from distal to proximal): fg, fenestrated glia; psug, pseudocartridge glia; dsg, distal satellite glia; psg, proximal satellite glia; eg, epithelial glia; mg: marginal glia. Scale bars: 20 µm (F, for A–F), 20 µm (H) and 5 µm (I).

Antibodies raised against the long (P1) or short (P2) isoform of Inebriated revealed a highly organized complementary distribution of each isoform within the lamina, with P1 expressed in the pigment cells and P2 in the photoreceptors (Fig. 1). The antibodies labelled a striking pattern of alternating layers of glia: (from distal to proximal, respectively) the fenestrated (P1), pseudocartridge (P2), distal (P1) and proximal (P2) satellite, epithelial (P1) and marginal (P2) glia. In the ommatidia all cells were immunolabelled with the P2 antibody, except for secondary and tertiary pigment cells, which were labelled with anti-P1 (Fig. 2).

**Fig. 3.**
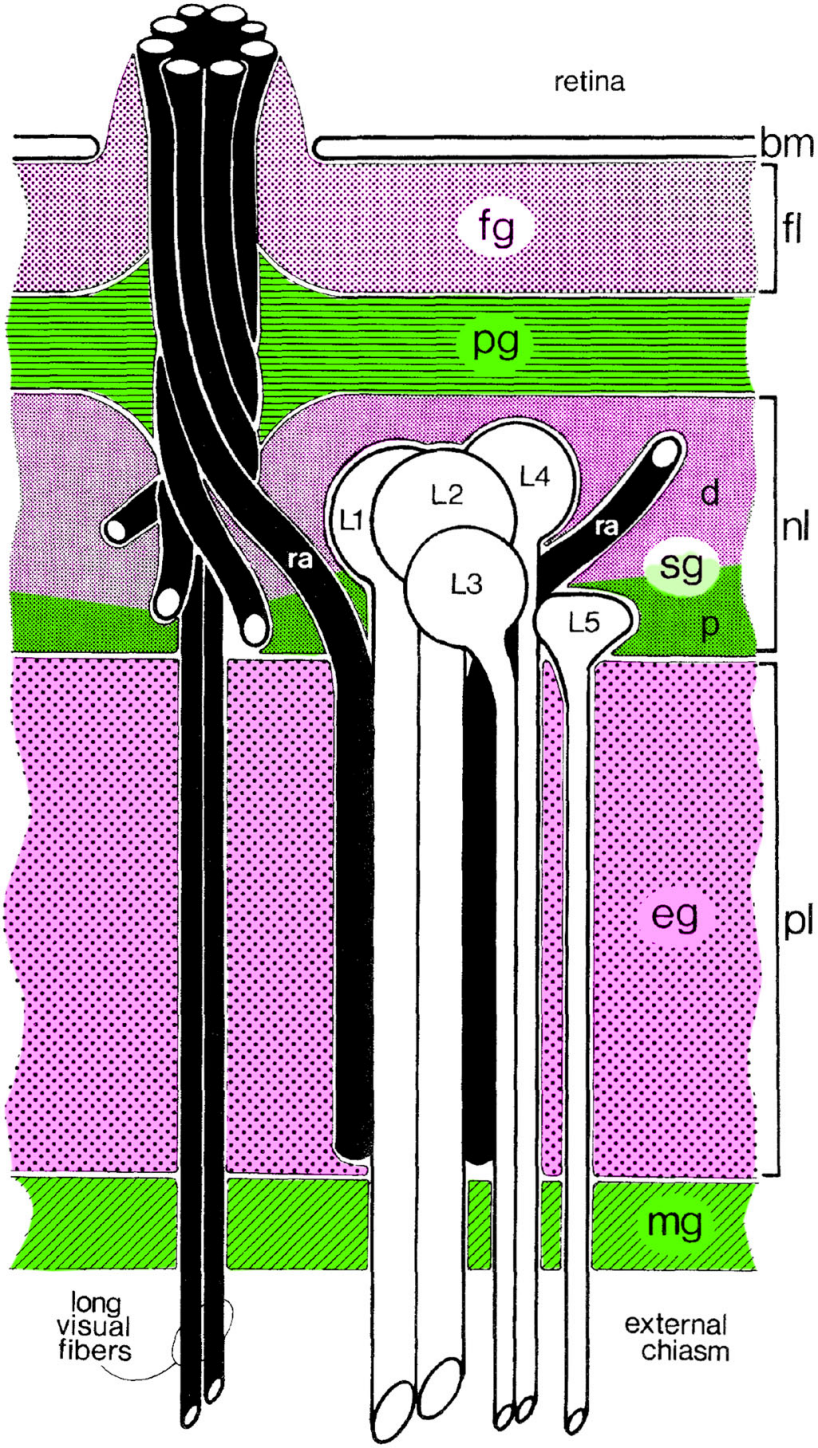
**Expression of the long (Ine-P1; magenta) and short (Ine-P2; green) isoforms of Inebriated in alternate layers of glia of the fly's optic lamina.** Ine-P1 is expressed in the fenestrated glia (fg), the distal (d) region of the satellite glia (sg), and the epithelial glia (eg). Ine-P2 is expressed in the pseudocartridge glia (pg), proximal (p) satellite glia (sg), and marginal glia (mg). Successive layers from distal to proximal are: the basement membrane (bm), fenestrated layer (fl), nuclear layer (nl), plexiform layer (pl) and photoreceptors axons (ra). Figure based on Saint Marie and Carlson (1983); the distinction between two layers of satellite glia (sg), proximal (p) and distal (d), was made according to Edwards and Meinertzhagen (2010).

**Fig. 4.**
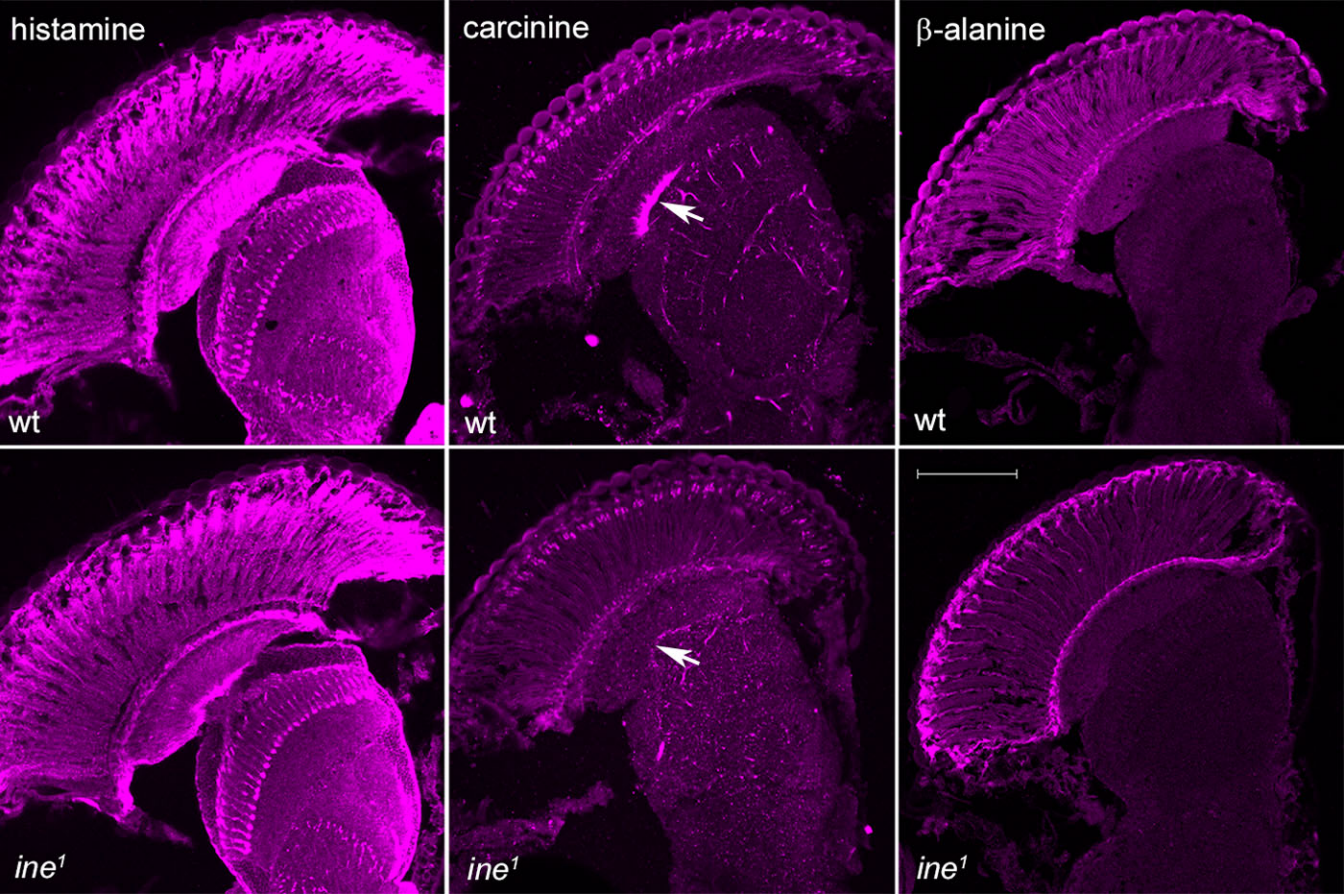
**Immunoreactivity to HA, CA and β-Ala are all reduced in specific cell types in *ine^1^* mutant flies compared with Oregon R wild type (wt), indicating the selective reductions in histamine and its two major metabolites.** The reduction was greatest in the lamina, and the difference most distinct with anti-carcinine labelling in the marginal glia (arrows). Scale bar: 50 µm (β-alanine in *ine^1^*).

Photoreceptors specifically express the P2 isoform whereas P1 is expressed in non-neuronal cells. Of three *ine* mutants, *ine^1^* and *ine^3^* are null for both isoforms whereas *ine^2^* does not express P1 but still produces P2 (Huang et al., 2002). Confirming antibody specificity, the *ine^1^* single mutant was immunolabelled with neither the P1 nor the P2 antibody (not shown). The alternating pattern of P1 and P2 expression in the depth of the optic lobe is summarized in Fig. 3. Relative to wild type, *ine^1^* flies show reduced HPLC signal for all three histamine-recycling components: HA, CA and β-Ala (Fig. 4). The major changes were visible in the lamina for anti-HA and anti-CA staining and in the retina for anti-β-Ala. In the wild type, HA was localized to photoreceptors and their terminals in the lamina and medulla and HA signal was reduced in *ine^1^*. In the wild type, anti-CA labelled primary pigment cells beneath the cornea, an area beneath the basement membrane, in the pseudocartridge and fenestrated glia, and underneath the lamina (mostly in the marginal glia). In the *ine^1^* mutant the signal was reduced especially in the marginal glia. In the wild type, anti-β-Ala labels photoreceptors especially beneath the cornea and along the basement membrane. This signal was reduced in *ine^1^* (Fig. 4).

### HPLC determinations

**Fig. 5.**
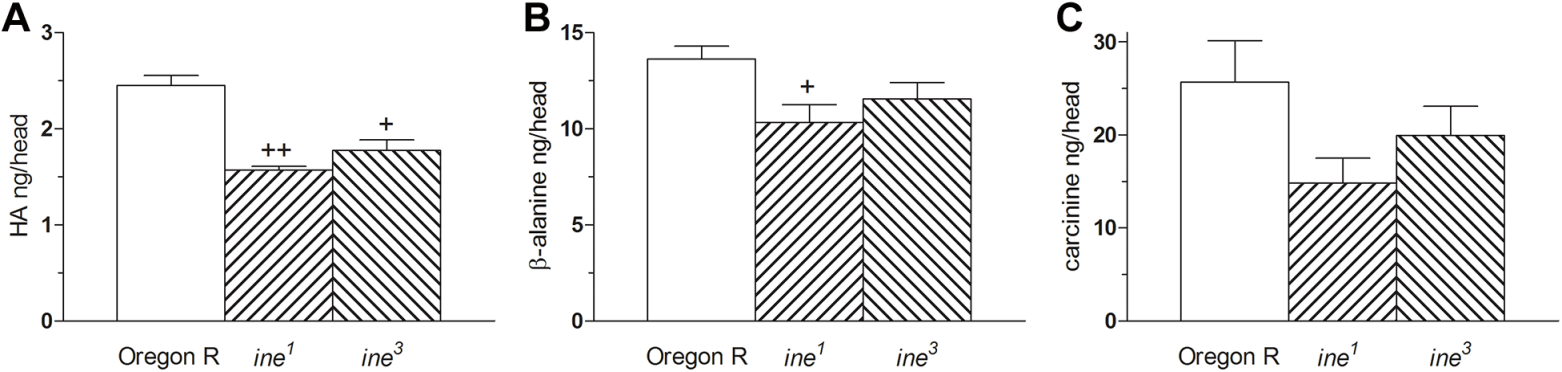
**Head HA (A), β-Ala (B) and CA (C) are all reduced in *ine^1^* and *ine^3^* mutants, compared with Oregon R wild-type flies (wt), confirming reductions in immunolabelling seen in** **Fig. 4**. Values differ statistically at *P*<0.05 (+) or *P*<0.01 (++), ANOVA followed by Tukey's HSD test (mean±s.d.; *n*=12/group).

**Fig. 6.**
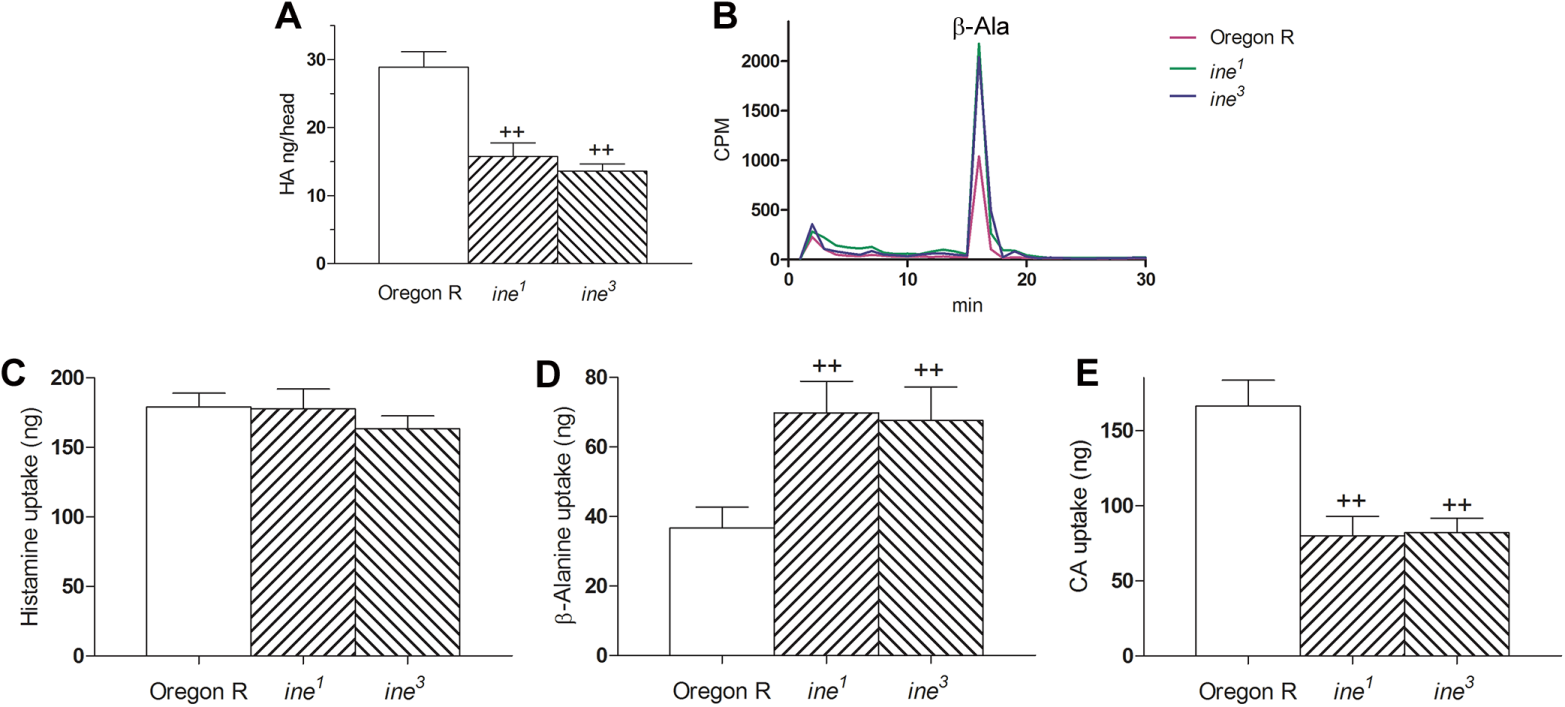
(A) Head HA content from flies after drinking solutions of carcinine (1%) suspended in 4% glucose for 12 h. The heads of both *ine^1^* and *ine^3^* mutant flies accumulate significantly less histamine after drinking the carcinine solution than Oregon R control wild-type flies (mean±s.d.; *n*=7/group). (B) Head accumulation of tritiated β-Alanine (β-Ala) after drinking for 40 min 25% [^3^H] β-alanine (37 MBq l^−1^ and 1850 GBq mmol^−1^ suspended in 4% glucose. Both *ine* mutants accumulate in their heads twice as much tritiated β-Ala as do Oregon R wild-type control flies (wt). Injected volume represents the radioactivity in a single head and is shown as counts per minute (CPM). (C–E) Fly synaptosome fractions incubated for 1 h in either 0.05% HA or 0.05% β-Ala or 0.1% CA suspended in fly saline (each group *n*=7). (C) The uptake of 0.05% HA into the synaptosomes of wt and *ine* mutant fly heads showed no differences; whereas (D) the uptake of 0.05% β-Ala was significantly increased in *ine* flies; and, in contrast (E), the uptake of 0.1% CA is strongly reduced in both *ine* mutants. (D–E) Values differ statistically at *P*<0.01 (++), ANOVA followed by Tukey's HSD test (C–E, mean±s.d.; *n*=7/group).

*ine^1^* and *ine^3^* mutants have a significantly reduced head HA content, which is 26% and 28%, respectively, less than in wild-type controls (Fig. 5A). Head β-alanine is significantly reduced by 24% in *ine^1^*, but less so (reduced by 15%) in *ine^3^* (Fig. 5B). Head carcinine is also reduced in *ine^1^* and *ine^3^* mutants, by 42% and 32% respectively (Fig. 5C). These findings strongly suggest that Inebriated may be involved in transporting HA and/or CA and β-Ala in the *Drosophila* eye. Indeed, the head contents of HA after drinking 0.1% CA for 12 h were reduced by 45% and 53% in *ine^1^* and *ine^3^* mutants, indicating that Inebriated may be involved in either CA or HA transport, or both, within the fly's head (Fig. 6A). Moreover, mutant *ine^1^* and *ine^3^* flies that drank a ^3^H-β-Ala solution for 40 min accumulated radioactive β-Ala at double the rate of wild-type controls, suggesting that Inebriated may be involved not only in transporting CA but also reciprocally transporting β-Ala, which accumulates rapidly in mutants given exogenous β-Ala (Fig. 6B). On the other hand, uptake of histamine into synaptosomes was not altered in either mutant, indicating that Inebriated, which is present in the synaptosomal preparation, does not mediate HA transport in those fractions when their Inebriated is mutant (Fig. 6C). Since synaptosomal preparations contain only trace amounts of glia (Borycz et al., 2008), changes in the uptake of histamine-recycling components are interpreted solely to reflect differences in neuronal uptake. As for *in vivo* studies, the *in vitro* uptake of β-Ala into the *ine* synaptosomes was also significantly increased (by 90% in *ine^1^* and by 84% in *ine^3^*) compared with wild-type synaptosomal uptake (Fig. 6D). Synaptosomal uptake of CA is reduced by 52% in *ine^1^* and by 51% in *ine^3^* (Fig. 6E). HA was taken up into synaptosomes approximately five-sixfold times more than β-Ala uptake, when both were applied at the same concentrations. This difference suggests that: (1) there is active transport of HA into the neuron; or (2) the β-Ala that enters the neuron is actively pumped by Ine and that this function is abolished in *ine* mutants. Altogether our results suggest that Inebriated may transport CA into neurons while reciprocally pumping β-Ala out of photoreceptors.

### The possible function of Ine expressed in *Xenopus* oocytes

**Fig. 7.**
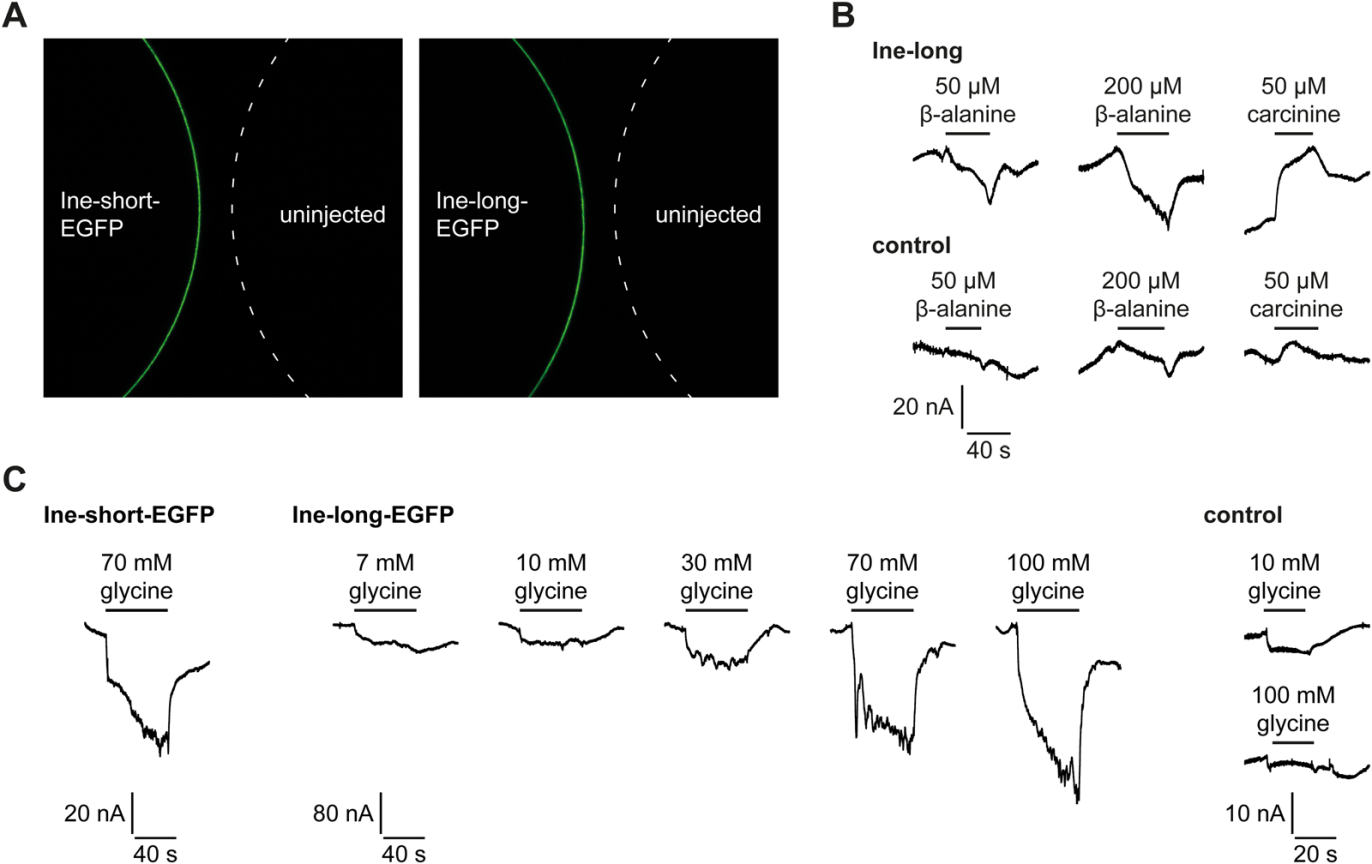
**Effects of β-Ala and CA on transmembrane conductance of *Xenopus laevis* oocytes transfected with Inebriated RNA.** (A) Oocytes expressing Ine-P1::GFP or Ine-P2::GFP on the left, next to uninjected control oocytes. (B,C) Current recordings from *Xenopus* oocytes expressing Ine-P1or water-injected controls held in solutions of β-alanine or carcinine at the given concentrations. (B) Representative traces show currents induced by applying various concentrations of β-alanine and carcinine, and glycine. (C) Glycine generates a significant current in oocytes transfected by transcripts for both the long (Ine-P1) and short (Ine-P2) Inebriated isoforms. Oocytes transfected by the transcript of the long (Ine-P1) protein isoform exhibit dose-dependent glycine currents over a range from 7 mM to 100 mM, relative to control oocytes (right).

To test for possible transport functions exerted by Ine, we transfected *Xenopus* oocytes with transcripts for the P1 and P2 Inebriated isoforms and measured transmembrane currents generated by the oocytes as the corresponding transmembrane conductance changes under current clamp conditions, using previously published methods (Tapken et al., 2013). Currents were measured in the presence of candidate transport substrates, of which we were most interested in the candidacy of β-Ala and CA. The number of successful recordings was quite small and some recordings were indistinct or not reproducible, giving no clear result overall. On the other hand, from a selection of several amino acids, the effect of different concentrations of glycine on Ine-P1-EGFP transfected oocytes revealed an obvious dose-dependent signal not shown by water-injected control oocytes, even for the few oocytes we were able to record in this way (Fig. 7; Table S1).

### Phototaxis

**Fig. 8.**
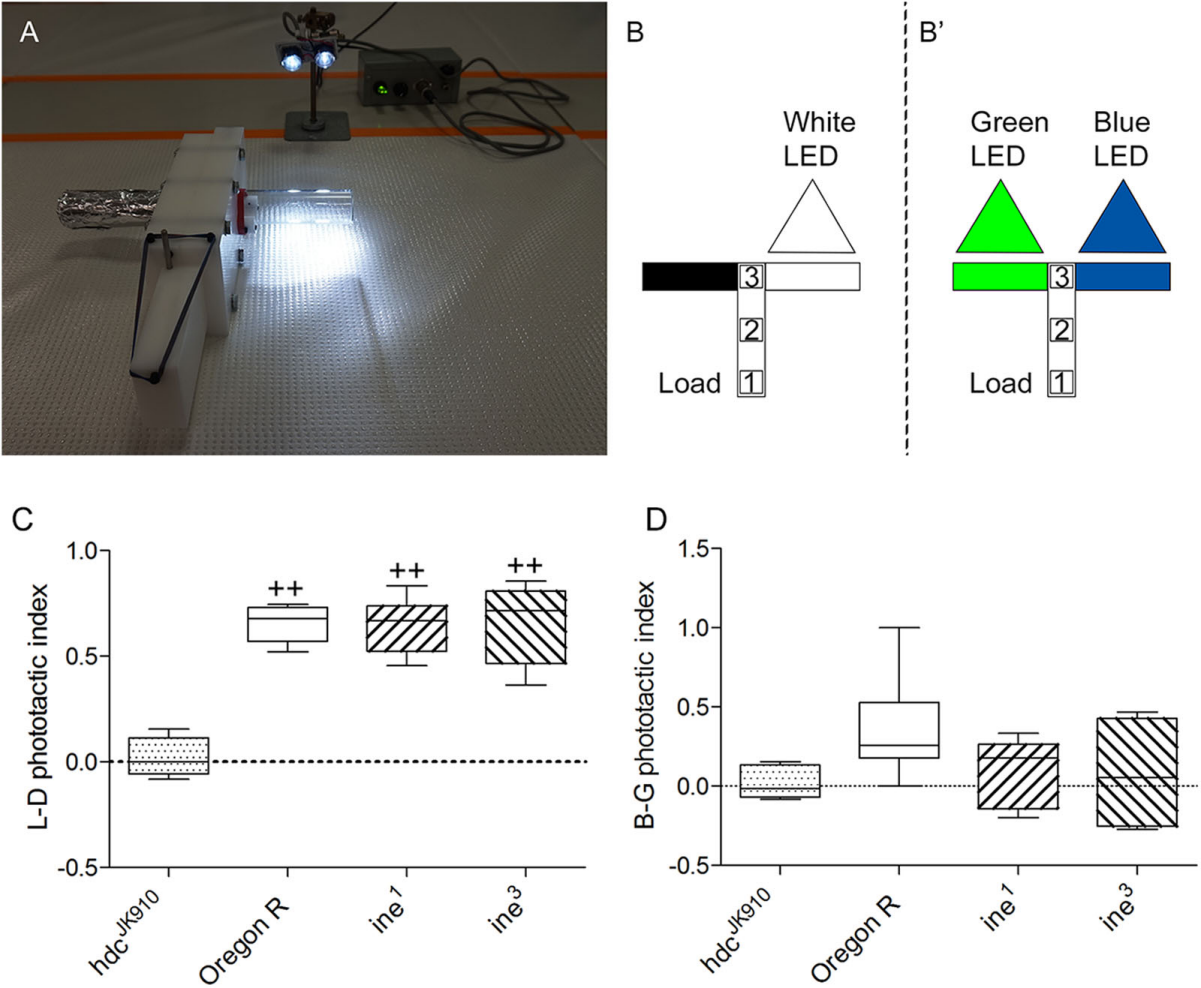
**Phototactic index for *ine* mutant *Drosophila* relative to *hdc* flies, which lack HA.** (A) Image of phototaxis assay equipment used to quantify *Drosophila* phototaxis in the light-dark paradigm. (B,B′) Diagram of the T-maze setups used to record phototaxis. In the light-dark paradigm (B) the lit arm (clear glass tube shown as an open rectangle) was illuminated by two blue-white LEDs (Luxeon Star/O, LXHK- WE8). The other tube (dark rectangle) was opaque. In the blue-green choice paradigm (B′) both tubes were clear, illuminated in one by four blue LEDs (467 nm; RL5-B55515 SuperbrightLeds) and in the other by four green LEDs (525 nm; RL5-G13008 SuperbrightLeds). Flies are manipulated in three slider positions of the specimen chamber: load (1), intermediate (2) and testing (3). The dimensions shown are not in proportion. (C) Wild-type OR flies show a strong preference for the lit arm, whereas most *hdc^JK910^* flies fail to move into either the lit or dark arm. L–D phototactic indices calculated from *ine* mutant performances differ significantly from blind *hdc^JK910^* flies, at *P*<0.01 (++) followed by Tukey's HSD test, but do not differ from Oregon R wild-type control flies in a light-dark paradigm. (D) *ine^1^* and *ine^3^* show a smaller preference for the blue-lit arm compared with wild type but the differences are not statistically significant. (C–D) mean±s.d.; *n*=8/group.

To examine whether *ine* mutant flies retained some capacity for vision, we next sought a visuomotor phenotype in *ine* mutant flies. In a light-dark choice, wild-type Oregon R flies mostly explored the lit arm (72%) with only 22% entering the dark arm, whereas 6% remained in the loading chamber (*n*=8 groups throughout). In a blue-green choice, 62% of wild type chose the blue-lit arm, 20% the green-lit arm and 18% remained in the loading chamber (*n*=8 groups throughout). In *hdc^JK910^*, which cannot synthesize HA and is reportedly blind (Burg et al., 1993; Melzig et al., 1996), 67% of flies remained in the loading chamber in a light-dark choice; only 17% and 16% explored either lit or dark arm, respectively. In a blue-green choice, the number of immobile *hdc^JK910^* flies increased to 85% and only 5% and 10% entered either green or blue arm, respectively. The difference in *hdc^JK91^* observed under two different illumination paradigms may indicate that despite the lack of a synthetic enzyme for HA in these flies they may nevertheless acquire it by taking up the amine from the gut microbiota or from the fly's food. With respect to vision in *ine* mutant flies, the performance index in *ine^1^* and *ine^3^* (Fig. 8) did not differ significantly from wild type, indicating that unlike *hdc,* and despite previous evidence from recordings of the electroretinogram (ERG) (Gavin et al., 2007), *ine* mutants are in fact able to detect light. The performance of *ine* in a blue-green choice was not the same as wild type, however, although the difference was insignificant, with roughly equal numbers of mutant flies entering either the blue- or the green-lit arm. Although insignificant, this small preference may nevertheless indicate that the fly's spectral perception or its preferences are disrupted by the *Inebriated* mutation.

## DISCUSSION

The most striking information from Inebriated P1 and P2 immunocytochemistry is the alternating expression pattern observed in contiguous glial layers in the lamina (Fig. 3). This pattern may indicate that morphologically different types of glia are also specialized to transport different substrates or possibly only a movement of ionic currents. A glial function for Inebriated is otherwise mostly not known. A previous study has demonstrated that Inebriated affects neuronal excitability when expressed either in neurons or in glia, and that each form of Inebriated is functional (Huang and Stern, 2002). Perineurial glial growth is also dependent on Inebriated (Yager et al., 2001). So too is systemic water homeostasis (Luan et al., 2015). Similar patterns of Ine expression, and functions, have also been observed in the *Inebriated* orthologue present in another insect species, *Manduca sexta* (Chiu et al., 2000). Most tantalizing is the organisation of Inebriated-P1 and -P2 glial expression in alternate layers of glial cells. We may propose that the significance of these is to ensure directionality in the flow of ions and/or substrate molecules between the alternating layers of Inebriated-P1- and -P2-expressing glial cell layers. Further interpretation must however await identification of the molecules and/or ions pumped by both Inebriated isoforms. It is not clear whether their combined actions might serve to maintain a barrier to the histamine that is released from photoreceptor terminals in the underlying lamina neuropile, nor, in that case, what function either isoform might have in the overlying retina. An alternative model would be if glial *Ine* expression were to maintain a barrier to the chloride ion, which becomes redistributed after the histamine, released from photoreceptor terminals, acts on *ort* receptors expressed by postsynaptic lamina cell target neurons (Pantazis et al., 2008).

In our observations we were unable to distinguish glial functions for Inebriated but we could dissect the neuronal uptake using synaptosome fractions, which contain mostly neuronal components. These clearly suggest involvement of Inebriated in neuronal uptake of CA and release of β-Ala. The accumulation of tritiated β-Ala in the head of *ine* mutants further supports this hypothesis as well as the significantly reduced head HA content, which is compatible with disrupted HA recycling in these mutants.

We have recently reported (Xu et al., 2015) that CarT is a transporter involved in carcinine recycling, a role previously suggested for Inebriated (Gavin et al., 2007). The role of CarT has been confirmed in recent reports (Stenesen et al., 2015; Chaturvedi et al., 2016). Our new results now indicate that Inebriated may be another protein with a candidate transport role for carcinine, but not directly as its specific transporter. Especially significant is that CarT expression is observed only in the photoreceptor’s terminals (Xu et al., 2015), whereas in the eye, CA expression is observed in the glia, where CarT does not express (Borycz et al., 2012). We note that the expression of multiple transporters carrying the same substrate is not unusual: for example, in vertebrates there are four known GABA transporters, together with one taurine and one creatinine transporter, which all arose by gene duplications from a single invertebrate GABA transporter (Kinjo et al., 2013). For glutamate in vertebrates there are five known subtypes of excitatory amino acid transporters that terminate the action of glutamate neurotransmitter at the synapse (Vandenberg and Ryan, 2013). This multiplicity suggests the possibility that the *Drosophila* eye may likewise express more than a single transporter involved in recycling histamine, a possibility that should also be taken into consideration. The requirement for that possibility is enhanced by the high rates of histamine release at fly photoreceptor terminals (de Ruyter van Steveninck and Laughlin, 1996; Stuart et al., 2007; Borycz et al., 2008).

Other evidence also supports the existence of multiple CA transporters. For example, Ziegler et al. (2013) have demonstrated that giving exogenous HA to double mutant *hdc^JK910^*; *e^AFA^* flies, which can synthesize neither HA nor CA, also fails to rescue the ‘on’ and ‘off’ transients of the electroretinogram (ERG), which report photoreceptor transmission in the lamina (Heisenberg, 1971). That finding indicates that conjugation of HA with β-Ala is necessary to transport the HA within the eye, a conclusion that would further support the hypothesis that multiple transporters of CA exist in the fruit fly's head.

SLC6 family transporters, which include Inebriated, may transport different amino acids reciprocally (Rudnick et al., 2014). Thus β-Ala is loaded into synaptic vesicles in rat brain preparations (Agullo et al., 1986; Fykse and Fonnum, 1996), while the presence of synaptic vesicles in the photoreceptors of *Drosophila* histamine-deficient flies mutant for *hdc* (J.B. and J.A.B., unpublished) suggests that β-Ala may instead be loaded into vesicles. To assess this possibility, we analysed the vesicular fraction of brain homogenates. Vesicle preparations of wild-type flies contain measurable amounts of histamine but no β-Ala, while vesicular fractions from the histamine-deficient mutant *hdc* contained neither HA nor β-Ala, further supporting our suggestion that Inebriated is the main transport mechanism to pump β-Ala out of photoreceptors. A transport role for Inebriated was not clearly revealed by our *Xenopus* oocyte expression studies, however, which if anything reveal that Ine may be involved in transporting glycine. However, in a related study (Chiu et al., 2000), uptake was also not observed with either construct of MasIne, the *Inebriated* homologue from *Manduca sexta*, using radiolabelled dopamine, serotonin, norepinephrine, octopamine, histamine, tyramine, glycine, GABA, glutamate, proline, lysine, phenylalanine, leucine, choline, taurine and creatine. It is unfortunate that neither β-Alanine nor carcinine was tested with MasIne, however, making the comparison incomplete. Moreover, our findings do suggest that glycine may be transported by Ine, whereas it was not with MasIne. Two other glycine transporter candidates are also reported, and one of these has been recently characterized in *Drosophila* (Frenkel et al., 2017).

In phototactic experiments our wild-type flies preferred the blue-lit arm of the T-maze, as previously reported (Jacob et al., 1977). However, *Drosophila*'s attraction to blue light has not always been observed and some studies report instead a green preference (Bosch et al., 2016). Regardless, the phototactic responses of *inebriated* suggest that this mutant is not, in fact, blind. This capacity is perhaps not surprising since there is a confirmed carcinine transporter, CarT, present in the eye, the function of which may compensate for the lack of Inebriated, for which the role remains uncertain. However, our results indicate that CarT alone is not sufficient to maintain wild-type colour vision in flies. Some fraction of histamine in *inebriated* photoreceptors must be synthesized anew from L-histidine and this amount will add to the neurotransmitter pool, release of which seems to be sufficient to distinguish the light source if not its specific wavelength.

In summary, our results fail to support any conclusion that Inebriated is involved directly in transporting CA into the photoreceptor neurons in the lamina, as previously proposed (Gavin et al., 2007), but a function for which the action of CarT is instead now confirmed (Xu et al., 2015). However, we cannot exclude that it has a supporting function in transporting CA in the eye's structures, where CarT is not expressed. The complementary additional role, the simultaneous transport of β-Ala in the opposite direction, remains unassigned to a candidate transporter. Similarly, as in previously published studies (Soehnge et al., 1996; Chiu et al., 2000; Huang and Stern, 2002), our data cannot clearly discern whether Inebriated itself is responsible for substrate transport or is only necessary to maintain the ionic gradient that drives that transport. Likewise, as for previously published data we have not been able to observe that Inebriated transports HA, and a photoreceptor HA transporter has therefore yet to be identified. Even so, our synaptosomal uptake studies do indicate that for all the components of a HA-recycling pathway (HA, CA and β-Ala), HA is the most efficiently transported, suggesting the presence of a specific HA transporter possibly in addition to indirect transport mechanisms. The search for a specific histamine transporter therefore remains the most significant target for future studies.

## MATERIALS AND METHODS

### Animals

Flies, *Drosophila melanogaster* Meigen, were held at 23°C on a standard cornmeal and molasses medium under a 12 h:12 h light:dark cycle. The following genotypes were used: (1) Oregon R (OR) wild type; (2) *ine^1^* (*inebriated^1^*) and *ine^3^* (*inebriated^3^*), both in an Oregon R background; and (3) Gal4 lines, as follows, which when crossed with UAS-mCD8::GFP drive the expression of GFP in distinct types of optic lobe glia (Edwards et al., 2012). We used the following Gal4 lines: *R43H01*; *R10D10*; *Mz97-GAL4*, *UAS-Stinger*; *R32 H04*; *R19CO2*; *gli^RL82^-GAL4*; and *R29A12*.

### Immunohistochemistry and confocal microscopy

For immunohistochemistry, heads were fixed in 4% formaldehyde (as paraformaldehyde), embedded in OCT (Sakura Finetechnical Co., Ltd, Tokyo, Japan), frozen in liquid nitrogen, and sections were cut in a frontal plane at 10 µm thickness on a cryostat (Reichert-Jung 2800, Frigocut: Leica Biosystems GmbH, Nussloch, Germany). Sections were processed for single or double immunolabelling using the following primary antibodies: β-alanine (at 1:1000) and/or β-alanylhistamine (carcinine, at 1:100). Polyclonal antibody (Abcam, catalogue no. ab37076-50, lot 700794) was raised in rabbit against β-alanine conjugated to BSA with glutaraldehyde; its specificity on *Drosophila* tissue was confirmed by the absence of immunolabelling after preadsorption with β-alanine, and by changes in signal after treatments reported in the Results. A rat polyclonal antibody was raised against a concatemer of β-alanyl-histamine conjugated to keyhole limpet haemocyanin (KLH). Its specificity in *Drosophila* was confirmed by us from the lack of signal after preadsorption with carcinine, but not after histamine or β-alanine, all at 10^–3^ to 10^–5^ mol l^–1^; immunolabelling was reduced in the mutant *ebony^1^* (data not shown). The following secondary antibodies were used: Cy-3-conjugated goat anti-rabbit (Jackson ImmunoResearch, West Grove, USA) at 1:400; Alexa Fluor 488 goat anti-mouse (Molecular Probes, Eugene, USA) at 1:100; and Alexa Fluor 488 goat anti-rat (Molecular Probes) at 1:100. Labelled sections were mounted in Vectashield and images were collected with Zeiss LSM 410 or 510 confocal microscopes, using Plan Neofluar 40/1.4 (LSM410), 40/1.3, 63/1.4 or 100/1.4 (LSM510) oil-immersion objectives. Images of preparations from wild-type and mutant heads, labelled with β-alanine, were collected using identical confocal operating parameters for brightness and contrast throughout.

### Anti-Ine antibody production

The N-terminus of Ine varies in both long (P1) and short (P2) isoforms. To generate a specific antiserum against the long Ine-P1 isoform we amplified the Ine-P1 specific N-terminal sequence using PCR and the following primers (including underlined EcoRI and HindIII sites respectively): GGATCCATATGGCGGAGAACAAAGACG and AAGCTTTGGTGGCGTCTGCGGATTG using a cDNA clone (HL05815, BDGP Berkeley, USA). The amplicon was subcloned into pGEMT Easy (Promega GmbH, Mannheim, Germany) and further cloned into the pQE 82L expression vector, which allows the generation of N-terminal His-tagged proteins (Qiagen GmbH, Hilden, Germany). The approximate 32.4 kDa N-terminal region of Ine-P1 was expressed in *E.coli*, affinity purified with Ni-TED resin (Macherey-Nagel GmbH, Düren, Germany), and injected into guinea pig (Eurogentec S.A., Seraing, Liège, Belgium). A second antibody to Ine-P1 was raised in rabbit using an HPLC-purified peptide H_2_N-MPNRQDYDAQSSKHS+ C-CONH_2,_ coupled to KLH.

### Uptake studies

(A) *In vivo*: flies were given solutions of carcinine (1%) suspended in 4% glucose to drink for 12 h, or were given 25% [^3^H] β-alanine (37 MBql^−1^ and 1850 GBq mmol l^−1^) suspended in 4% glucose, for 40 min. For processing of [^3^H] β-alanine samples, see below under ‘Uptake of tritiated β-alanine’.

(B) *In vitro*: we made fly synaptosome fractions, and incubated these for 1 h in either 0.05% HA or 0.05% β-Ala or 0.1% CA suspended in fly saline. The fractions were prepared according to the following procedure: 500–600 heads were cut from flies using a razor blade and kept on ice in a Petri dish, and were then homogenized in 2 ml of ice-cold Ringer's, followed by centrifugation for 10 min at 4°C and 2000* **g***. The pellet was discarded, and the supernatant centrifuged again for 20 min, at 4°C and 20,000* **g***. The Ringer's contained (in mM) NaCl 127, glucose 55.5, KCl 5, CaCl_2_ 2.7, Tris 1.25, EDTA 1, MgSO_4_ 0.65, and one tablet of Roche Complete mini (protease inhibitor) per 50 ml of Ringer's was added prior to homogenization.

### High-performance liquid chromatography (HPLC)

To determine brain histamine, β-alanine and carcinine, flies were collected, frozen at −80°C, shaken to decapitate them, and the heads then processed using HPLC with electrochemical detection, all as previously reported (Borycz et al., 2000). In this method, CA, β-alanine, HA and 3-methylhistamine (as an internal standard) were clearly separated with respective retention times at roughly 11 min, 15 min, 18 min and 21 min. The carcinine determinations sometimes showed an unidentified overlapping peak having a similar retention time, and determinations from such peaks were disregarded. We used samples from 50 *Drosophila* heads, and calculated the mean of the mean values from between 7 and 12 such samples.

### Uptake of tritiated β-alanine

The method was adapted from our previous report (Borycz et al., 2002). Flies were dehydrated for 3 h, after which they were given a droplet to drink of 25% [^3^H]β-alanine (37 MBq ml^–1^ and 1850 GBq mmol l^–1^; American Radiolabeled Chemicals Inc., St Louis, USA) in 4% aqueous glucose. After 40 min, the flies were frozen, and their heads collected and prepared for HPLC as above. Samples were separated by HPLC, and fractions of the mobile phase collected at 1 min intervals. Samples of the mobile phase, 1 ml mixed with 5 ml of scintillation cocktail (Ready Safe; Beckman Coulter, Mississauga, Canada), were counted for 5 min in a scintillation counter (Beckman Coulter LS 6500). The retention time for [^3^H]β-alanine in these fractions was confirmed exactly from the retention time for the cold β-alanine peaks obtained by electrochemical detection.

### Oocyte expression studies

cRNA was synthesized from 0.7 µg of linearized plasmid DNA using the mMESSAGE mMACHINE T7 kit (Thermo Fisher Scientific). The template DNA was digested using DNase and the RNA purified by phenol extraction. Oocyte injection and voltage clamp recordings were performed as previously reported (Tapken et al., 2013). Briefly, oocytes were surgically removed from *Xenopus laevis* (Nasco, Fort Atkinson, USA) and defolliculated with 784 U/ml (4 mg/ml) collagenase type I (Worthington, Lakewood, USA) in Ca^2+^-free Barth's solution for 2 h at 20°C. After washing with Barth's solution, stage V or VI oocytes were selected, injected with 20 ng (50 nl) of cRNA or 50 nl of water (negative controls) using a nanolitre injector (WPI, Sarasota, USA), and then maintained at 16°C in Barth's solution, supplemented with antibiotics. 4–6 days after injection, current responses were recorded under voltage clamp at −100 mV with a Turbo Tec-10CD amplifier (npi electronic GmbH, Tamm, Germany) controlled by Patchmaster software (HEKA, Lambrecht, Germany). Recordings were undertaken in normal frog Ringer's solution (NFR) in a 50 µl chamber. 100 µl of the analysed substance dissolved in NFR was added directly to the recording chamber with a pipette and removed after 20–60 s by rinsing with NFR at a flow rate of 3–5 ml/min.

### Phototaxis

A T-maze apparatus for phototaxis designed by Mr Hunter Shaw, McGill University, was used in one of two visuomotor paradigms, either a light-dark choice or a blue-green choice. In the light/dark choice, the lit arm (a clear glass tube) was illuminated by two blue-white LEDs (Luxeon Star/O, LXHK-WE8). The other tube was opaque. In the blue/green choice, four LEDs of 467 nm and four of 525 nm (RL5-B55515 or RL5-G13008 SuperbrightLeds, respectively) were used to illuminate the glass tubes. Unanaesthetized flies were loaded quickly into the apparatus, spent 60 s in the intermediate compartment, and were then moved to the open compartment with a light/dark choice, and remained for 30 s to reveal a phototactic decision. After 30 s the fly compartment was moved back to the intermediate position and flies were anaesthetized with CO_2_ and counted. A phototactic index adapted and modified from Gorostiza et al. (2016) was derived. Depending on the method of illumination this was calculated as either:

LD phototactic index=(no. of flies in the lit arm–no. of flies in the dark arm)/total no. of flies, or;

BG phototactic index=(no. of flies in the blue-lit arm–no. of flies in the green-lit arm)/total no. of flies.

### Statistical analyses

Throughout, values of biogenic amines are expressed as the mean±s.d. of the mean values for 7–12 independent samples of head amine determinations, or for one or two independent samples of ^3^H counts. To compare head contents of carcinine, β-alanine and histamine between wild-type and mutant flies, we used ANOVA followed by a Tukey's HSD test, by means of Systat 5.2.1 software (Systat, Chicago, USA).

## Supplementary Material

Supplementary information

## References

[BIO034926C1] Agullo L., Jimenez B., Aragón C. and Giménez C. (1986). β Alanine transport in synaptic plasma membrane vesicles from rat brain. *Europ. J. Biochem.* 159, 611-617. 10.1111/j.1432-1033.1986.tb09929.x 3093232

[BIO034926C3] Aust S., Brüsselbach F., Pütz S. and Hovemann B. T. (2010). Alternative tasks of *Drosophila* Tan in neurotransmitter recycling versus cuticle sclerotization disclosed by kinetic properties. *J. Biol. Chem.* 285, 20740-20747. 10.1074/jbc.M110.120170 20439462 PMC2898368

[BIO034926C2] Borycz J., Vohra M., Tokarczyk G. and Meinertzhagen I. A. (2000). The determination of histamine in the *Drosophila* head. *J. Neurosci. Methods* 101, 141-148. 10.1016/s0165-0270(00)00259-4 10996374

[BIO034926C4] Borycz J., Borycz J. A., Loubani M. and Meinertzhagen I. A. (2002). *tan* and *ebony* genes regulate a novel pathway for transmitter metabolism at fly photoreceptor terminals. *J. Neurosci.* 22, 10549-10557. 10.1523/JNEUROSCI.22-24-10549.2002 12486147 PMC6758454

[BIO034926C5] Borycz J., Borycz J. A., Kubów A., Lloyd V. and Meinertzhagen I. A. (2008). *Drosophila* ABC transporter mutants *white*, *brown* and *scarlet* have altered contents and distributions of biogenic amines in the brain. *J. Exp. Biol.* 211, 3454-3466. 10.1242/jeb.021162 18931318

[BIO034926C6] Borycz J., Borycz J. A., Edwards T., Boulianne G. L. and Meinertzhagen I. A. (2012). The metabolism of histamine in the *Drosophila* optic lobe involves an ommatidial pathway: β-alanine recycles through the retina. *J. Exp. Biol.* 215, 1399-1411. 10.1242/jeb.060699 22442379 PMC3309881

[BIO034926C7] Bosch D. S., van Swinderen B. and Millard S. S. (2016). *Dscam2* affects visual perception in *Drosophila melanogaster*. *Front. Behav. Neurosci.* 9, 149 10.3389/fnbeh.2015.00149 PMC4460526 26106310

[BIO034926C9] Burg M. G., Sarthy P. V., Koliantz G. and Pak W. L. (1993). Genetic and molecular identification of a *Drosophila* histidine decarboxylase gene required in photoreceptor transmitter synthesis. *EMBO J.* 12, 911-919. 8096176 10.1002/j.1460-2075.1993.tb05732.x PMC413291

[BIO034926C10] Burg M. G., Geng C., Guan Y., Koliantz G. and Pak W. L. (1996). *Drosophila rosA* gene, which when mutant causes aberrant photoreceptor oscillation, encodes a novel neurotransmitter transporter homologue. *J. Neurogenet.* 11, 59-79. 10.3109/01677069609107063 10876650

[BIO034926C11] Chaturvedi R., Luan Z., Guo P. and Li H.-S. (2016). *Drosophila* vision depends on carcinine uptake by an organic cation transporter. *Cell Rep.* 14, 2076-2083. 10.1016/j.celrep.2016.02.009 26923590 PMC4785057

[BIO034926C12] Chiu C. S., Ross L. S., Cohen B. N., Lester H. A. and Gill S. S. (2000). The transporter-like protein Inebriated mediates hyperosmotic stimuli through intracellular signaling. *J. Exp. Biol.* 203, 3531-3546. 11060215 10.1242/jeb.203.23.3531

[BIO034926C14] de Ruyter van Steveninck R. R. and Laughlin S. B. (1996). The rate of information transfer at graded-potential synapses*.* *Nature* 379, 642-645. 10.1038/379642a0

[BIO034926C15] Edwards T. N. and Meinertzhagen I. A. (2010). The functional organisation of glia in the adult brain of *Drosophila* and other insects. *Prog. Neurobiol.* 90, 471-497. 10.1016/j.pneurobio.2010.01.001 20109517 PMC2847375

[BIO034926C16] Edwards T. N., Nuschke A. C., Nern A. and Meinertzhagen I. A. (2012). Organization and metamorphosis of glia in the *Drosophila* visual system. *J. Comp. Neurol.* 520, 2067-2085. 10.1002/cne.23071 22351615

[BIO034926C17] Frenkel L., Muraro N. I., Beltrán González A. N., Marcora M. S., Bernabó G., Hermann-Luibl C., Romero J. I., Helfrich-Förster C., Castaño E. M., Marino-Busjle C. et al. (2017). Organization of circadian behavior relies on glycinergic transmission. *Cell Reports* 19, 72-85. 10.1016/j.celrep.2017.03.034 28380364

[BIO034926C18] Fykse E. M. and Fonnum F. (1996). Amino acid neurotransmission: dynamics of vesicular uptake. *Neurochem. Res.* 21, 1053-1060. 10.1007/BF02532415 8897468

[BIO034926C19] Gavin B. A., Arruda S. E. and Dolph P. J. (2007). The role of carcinine in signaling at the *Drosophila* photoreceptor synapse. *PLoS Genet.* 3, e206 10.1371/journal.pgen.0030206 18069895 PMC2134947

[BIO034926C20] Gorostiza E. A., Colomb J. and Brembs B. (2016). A decision underlies phototaxis in an insect. *Open Biol.* 6, 160229 10.1098/rsob.160229 28003472 PMC5204122

[BIO034926C21] Hardie R. C. (1987). Is histamine a neurotransmitter in insect photoreceptors? *J. Comp. Physiol. A* 161, 201-213. 10.1007/BF00615241 2442380

[BIO034926C22] Heisenberg M. (1971). Separation of receptor and lamina potentials in the electroretinogram of normal and mutant *Drosophila**.* *J. Exp. Biol.* 55, 85-100. 5001616 10.1242/jeb.55.1.85

[BIO034926C23] Hotta Y. and Benzer S. (1969). Abnormal electroretinograms in visual mutants of *Drosophila*. *Nature* 222, 354-356. 10.1038/222354a0 5782111

[BIO034926C24] Huang Y. and Stern M. (2002). In vivo properties of the *Drosophila inebriated*-encoded neurotransmitter transporter. *J. Neurosci.* 22, 1698-1708. 10.1523/JNEUROSCI.22-05-01698.2002 11880499 PMC6758900

[BIO034926C25] Huang X., Huang Y., Chinnappan R., Bocchini C., Gustin M. C. and Stern M. (2002). The *Drosophila inebriated*-encoded neurotransmitter/osmolyte transporter: dual roles in the control of neuronal excitability and the osmotic stress response. *Genetics* 160, 561-569. 11861562 10.1093/genetics/160.2.561 PMC1461969

[BIO034926C26] Jacob K. G., Willmund R., Folkers E., Fischbach K. F. and Spatz H.-C. H. (1977). T-maze phototaxis of *Drosophila melanogaster* and several mutants in the visual systems. *J. Comp. Physiol.* 116, 209-225. 10.1007/BF00605403

[BIO034926C27] Kinjo A., Koito T., Kawaguchi S. and Inoue K. (2013). Evolutionary history of the GABA transporter (GAT) group revealed by marine invertebrate GAT-1. *PLoS ONE* 8, e82410 10.1371/journal.pone.0082410 24312660 PMC3849432

[BIO034926C28] Luan Z., Quigley C. and Li H.-S. (2015). The putative Na^+^/Cl^−^-dependent neurotransmitter/osmolyte transporter inebriated in the *Drosophila* hindgut is essential for the maintenance of systemic water homeostasis. *Sci. Rep.* 5, 7993 10.1038/srep07993 25613130 PMC4303880

[BIO034926C30] Melzig J., Buchner S., Wiebel F., Wolf R., Burg M., Pak W. L. and Buchner E. (1996). Genetic depletion of histamine from the nervous system of *Drosophila* eliminates specific visual and mechanosensory behavior. *J. Comp. Physiol. A* 179, 763-773. 10.1007/BF00207355 8956497

[BIO034926C31] Pantazis A., Segaran A., Liu C.-H., Nikolaev A., Rister J., Thum A. S., Roeder T., Semenov E., Juusola M. and Hardie R. C. (2008). Distinct roles for two histamine receptors (*hclA* and *hclB*) at the *Drosophila* photoreceptor synapse. *J. Neurosci.* 28, 7250-7259. 10.1523/JNEUROSCI.1654-08.2008 18632929 PMC6670387

[BIO034926C32] Richardt A., Rybak J., Störtkuhl K. F., Meinertzhagen I. A. and Hovemann B. T. (2002). Ebony protein in the *Drosophila* nervous system: optic neuropile expression in glial cells. *J. Comp. Neurol.* 452, 93-102. 10.1002/cne.10360 12205712

[BIO034926C33] Rudnick G., Krämer R., Blakely R. D., Murphy D. L. and Verrey F. (2014). The SLC6 transporters: perspectives on structure, functions, regulation, and models for transporter dysfunction. *Pflügers Arch.* 466, 2542 10.1007/s00424-013-1410-1 PMC3930102 24337881

[BIO034926C34] Saint Marie R. L. and Carlson S. D. (1983). Glial membrane specializations and the compartmentalization of the lamina ganglionaris of the housefly compound eye. *J. Neurocytol.* 12, 243-275. 10.1007/BF01148464 6842276

[BIO034926C35] Sarthy P. V. (1989). Histamine: a neurotransmitter candidate for photoreceptors for *Drosophila melanogaster*. *J. Neurochem.* 57, 1757-1768. 10.1111/j.1471-4159.1991.tb06378.x 1717657

[BIO034926C36] Soehnge H., Huang X., Becker M., Whitley P., Conover D. and Stern M. (1996). A neurotransmitter transporter encoded by the *Drosophila inebriated* gene. *Proc. Natl. Acad. Sci. USA* 93, 13262-13267. 10.1073/pnas.93.23.13262 8917579 PMC24081

[BIO034926C37] Stenesen D., Moehlman A. T. and Krämer H. (2015). The carcinine transporter CarT is required in *Drosophila* photoreceptor neurons to sustain histamine recycling. *eLife* 4, e10972 10.7554/eLife.10972 26653853 PMC4739767

[BIO034926C38] Stern M. and Ganetzky B. (1992). Identification and characterization of *inebriated*, a gene affecting neuronal excitability in *Drosophila*. *J. Neurogenet.* 8, 157-172. 10.3109/01677069209083445 1334137

[BIO034926C39] Stuart A. E., Borycz J. and Meinertzhagen I. A. (2007). The dynamics of signaling at the histaminergic photoreceptor synapse of arthropods. *Progr. Neurobiol.* 82, 202-227. 10.1016/j.pneurobio.2007.03.006 17531368

[BIO034926C40] Tapken D., Anschütz U., Liu L. H., Huelsken T., Seebohm G., Becker D. and Hollmann M. (2013). A plant homolog of animal glutamate receptors is an ion channel gated by multiple hydrophobic amino acids. *Sci. Signal.* 6, ra47 10.1126/scisignal.2003762 23757024

[BIO034926C41] Vandenberg R. J. and Ryan R. M. (2013). Mechanisms of glutamate transport. *Physiol. Rev.* 93, 1621-1657. 10.1152/physrev.00007.2013 24137018

[BIO034926C42] Xu Y., An F., Borycz J. A., Borycz J., Meinertzhagen I. A. and Wang T. (2015). Histamine recycling is mediated by CarT, a carcinine transporter at the *Drosophila* photoreceptor synapse. *PLoS Genet.* 15, e1005764 10.1371/journal.pgen.1005764.eCollection2015 PMC4694695 26713872

[BIO034926C43] Yager J., Richards S., Hekmat-Scafe D. S., Hurdi D. D., Sundaresan V., Caprette D. R., Saxtoni W. M., Carlson J. R. and Stern M. (2001). Control of *Drosophila* perineurial glial growth by interacting neurotransmitter-mediated signaling pathways. *Proc. Natl Acad. Sci. USA* 98, 10445-10450. 10.1073/pnas.191107698 11517334 PMC56980

[BIO034926C44] Ziegler A. B., Brüsselbach F. and Hovemann B. T. (2013). Activity and coexpression of *Drosophila black* with *ebony* in fly optic lobes reveals putative cooperative tasks in vision that evade electroretinographic detection. *J. Comp. Neurol.* 521, 1207-1224. 10.1002/cne.23247 23124681

