# Supplementary material for: Location and functions of Inebriated in the Drosophila eye
Source: Biol Open. 2018 Jul 15;7(7):bio034926. doi: 10.1242/bio.034926 (PMC6078345; doi:10.1242/bio.034926)
Supplement: Supplementary information [file biolopen-7-034926-s1.pdf]

Biology Open (2018): doi:10.1242/bio.034926: Supplementary information

Supplementary Data S1. Xenopus electrophysiology data

Click here to Supplementary Data S1

Disease Models & Mechanisms • DMM • Accepted manuscript
